# Bacterial associates of *Orthezia urticae*, *Matsucoccus pini*, and *Steingelia gorodetskia* - scale insects of archaeoccoid families Ortheziidae, Matsucoccidae, and Steingeliidae (Hemiptera, Coccomorpha)

**DOI:** 10.1007/s00709-019-01377-z

**Published:** 2019-04-17

**Authors:** Katarzyna Michalik, Teresa Szklarzewicz, Małgorzata Kalandyk-Kołodziejczyk, Anna Michalik

**Affiliations:** 1 Department of Developmental Biology and Morphology of Invertebrates, Institute of Zoology and Biomedical Research, Faculty of Biology, Jagiellonian University, Gronostajowa 9, 30-387 Kraków, Poland; 2 Department of Zoology, Faculty of Biology and Environmental Protection, University of Silesia, Bankowa 9, 40-007 Katowice, Poland

**Keywords:** Symbiotic microorganisms, *Sphingomonas*, *Sodalis*-like symbionts, *Wolbachia*, Scale insects, Transovarial transmission

## Abstract

The biological nature, ultrastructure, distribution, and mode of transmission between generations of the microorganisms associated with three species (*Orthezia urticae*, *Matsucoccus pini*, *Steingelia gorodetskia*) of primitive families (archaeococcoids = Orthezioidea) of scale insects were investigated by means of microscopic and molecular methods. In all the specimens of *Orthezia urticae* and *Matsucoccus pini* examined, bacteria *Wolbachia* were identified. In some examined specimens of *O. urticae*, apart from *Wolbachia*, bacteria *Sodalis* were detected. In *Steingelia gorodetskia*, the bacteria of the genus *Sphingomonas* were found. In contrast to most plant sap-sucking hemipterans, the bacterial associates of *O. urticae*, *M. pini*, and *S. gorodetskia* are not harbored in specialized bacteriocytes, but are dispersed in the cells of different organs. Ultrastructural observations have shown that bacteria *Wolbachia* in *O. urticae* and *M. pini*, *Sodalis* in *O. urticae*, and *Sphingomonas* in *S. gorodetskia* are transovarially transmitted from mother to progeny.

**Electronic supplementary material:**

The online version of this article (10.1007/s00709-019-01377-z) contains supplementary material, which is available to authorized users.

## Introduction

Scale insects (coccoids) constitute the infraorder Coccomorpha within the hemipteran suborder Sternorrhyncha (Williams and Hodgson 2014). There are estimated to be about 8000 species of scale insects. Many coccoid species are considered to be economically important pests in horticulture, agriculture, and forestry (Gullan and Cook 2007; Gullan and Martin 2009). Scale insects are more diverse in terms of their morphology, chromosome systems, modes of reproduction (parthenogenesis, hermaphroditism, bisexual reproduction), and types of symbioses than any of the other sternorrhynchan groups (Gullan and Martin 2009).

Scale insects are usually divided into two groups: Orthe*z*ioidea Amyot et Serville, 1843 and Coccoidea Fallen, 1814 which are considered as superfamilies (Koteja 1974; Danzig 1980; Williams and Watson 1990; Morales 1991; Kosztarab 1996; Ben-Dov 2005; Gavrilov-Zimin 2018). Other researchers treat these groups as informal archaeococcoids and neococcoids (Cook et al. 2002; Foldi 2005; Hodgson and Foldi 2006; Hardy et al. 2008; Kaydan and Kozár 2010; Williams et al. 2011; Hodgson 2012, 2014; Hodgson and Hardy 2013). In the present work, we have investigated symbiotic systems of three species of archaeococcoids (= Orthe*z*ioidea): *Orthezia urticae* (Linnaeus, 1758), *Matsucoccus pini* (Green, 1925), and *Steingelia gorodetskia* Nasonov, 1908.

*Orthezia urticae* is a species which belongs to the family Ortheziidae, which has been considered to be one of the oldest families of scale insects (Koteja 1996; Kozár and Miller 2000; Vea and Grimaldi 2012).

The genera *Matsucoccus* Cockerell 1909 and *Steingelia* Nasonov 1908 have been ascribed to the family Margarodidae (e.g., Morrison 1928; Kosztarab and Kozár 1988; Ben-Dov 2005; Kozár et al. 2013; Gavrilov-Zimin 2018). Koteja (1974) proposed a phylogeny and classification of the scale insects that gave family rank to a number of groups that were previously placed within Margarodidae sensu lato, e.g., Matsucoccidae. The results of studies conducted by several authors have supported Koteja’s classification (e.g., Foldi 2004, 2005; Booth and Gullan 2006; Hodgson and Foldi 2006; Ben-Dov 2012; Hodgson and Hardy 2013; Mech et al. 2013; Wang et al. 2016). The genus *Steingelia* was initially placed in the family Kuwaniidae (Koteja 1974; Dziedzicka 1977), but was later assigned its own family, Steingeliidae (e.g., Koteja 1996, 2000).

Most scale insects, like many other hemipterans which feed on plant sap that is lacking essential amino acids, are host to obligate symbiotic microorganisms (reviewed in Buchner 1965; Tremblay 1977; Douglas 1989, 1998; Baumann 2005; Rosenblueth et al. 2018). Previous studies based on paraffin technique have revealed that scale insects, as opposed to remaining Sternorrhyncha (aphids, whiteflies, psyllids), are characterized by an enormous diversity of symbiotic associates (reviewed in Walczuch 1932; Buchner 1965; Tremblay 1977). More recent ultrastructural and molecular analyses have confirmed that the symbioses of scale insects are much more diverse than those in the remaining Sternorrhyncha, with respect to the systematic affiliation of symbionts, distribution in the host insect body, and the mode of transmission from the mother to the progeny (Fukatsu and Nikoh 2000; von Dohlen et al. 2001; Thao et al. 2002; Szklarzewicz et al. 2006, 2013, 2018; Niżnik and Szklarzewicz 2007; Kono et al. 2008; Matsuura et al. 2009; Gruwell et al. 2010, 2012; Ramirez-Puebla et al. 2010; Gatehouse et al. 2011; McCutcheon and von Dohlen 2011; Vashishtha et al. 2011; Dhami et al. 2012; Rosenblueth et al. 2012, 2018; Husnik et al. 2013; Koga et al. 2013a; Sabree et al. 2013; Rosas-Pérez et al. 2014; Michalik et al. 2016, 2018; Szabo et al. 2017). Several families of scale insects, e.g., Steingeliidae, Xylococcidae, Matsucoccidae, Kermesidae, Kuwaniidae, Dactylopiidae, were regarded as asymbiotic (Buchner 1965; Tremblay 1977); however, the results of recent ultrastructural or molecular studies have revealed that some of them, i.e., Steingeliidae, Dactylopiidae, Kermesidae, and Matsucoccidae, may harbor bacterial or yeast-like associates (Koteja et al. 2003; Ramirez-Puebla et al. 2010; Szklarzewicz et al. 2014; Podsiadło et al. 2018; Rosenblueth et al. 2018). In contrast to the neococcoids, which were the object of numerous molecular analyses, the symbiotic systems of archaeococcoids are not well known. Taking the above into consideration, the aim of the present study was to re-examine the representatives of the archaeococcoid families Ortheziidae, Steingeliidae, and Matsucoccidae and provide information on their microbiota.

## Material and methods

### Insects

*Orthezia urticae* (Linnaeus, 1758) (Fig. S1a) is a polyphagous pest of herbaceous plants which prefers the stinging nettle *Urtica dioica*. *O. urticae* develops one generation yearly (Kosztarab and Kozár 1988). The adult females of *O. urticae* were collected from the stems of *U. dioica* in May and June of the years 1994, 1995, 2015, and 2016 in Kraków (located in the south of Poland).

*Steingelia gorodetskia* Nasonov, 1908 (Fig. S1b) is a monophagous species which resides on the roots of birch trees. The life cycle of *S. gorodetskia* lasts 1 year (Koteja and Żak-Ogaza 1981). The larvae live on roots situated about 20 cm below the ground’s surface. Adult females migrate to the ground’s surface, where they lay eggs. The larvae of the last instar of *S. gorodetskia* were collected from the roots of the birch *Betula verrucosa* in April 2004 in Kraków. The adult females were collected from dry, fallen leaves of the birch *B. verrucosa* in May and June 2001, 2002, 2004, and 2016–2018 in Kraków and May and June 2016 in Kobiór (both localities in the southern region of Poland).

*Matsucoccus pini* (Green, 1925) (Fig. S1c), like other species of Matsucoccidae family, is a serious pest for pine forests (Foldi 2004). *M. pini* develops two generations a year (Siewniak 1976). The females of *M. pini* were collected from the bark crevices of the pine *Pinus sylvestris* in May 2012 and 2013 in Kuźnia Raciborska (located in the south of Poland).

### Molecular analyses

The specimens of *S. gorodetskia*, *M. pini*, and *O. urticae* designated for molecular analyses were fixed in 100% ethanol. Before DNA extraction, the specimens were placed in 5% sodium hypochlorite for 1 min and then rinsed in distillated water three times for 1 min. DNA was isolated from 20 individuals of *S. gorodetskia*, 20 of *O. urticae*, and 20 of *M. pini* using Sherlock AX extraction kit (A&A Biotechnology) abiding by manufacturer protocol. The identification of bacterial associates of the species examined was done on the basis of the sequences of their 16S rRNA genes. The 16S rRNA gene was amplified using universal, eubacterial primers: 8F and 1541R, and following this, the purified products were cloned into pJET1.2/blunt plasmid vector using a Clone JET PCR Cloning Kit (Thermo Scientific). The ligated mixtures were transformed into component cells *Escherichia coli* TOP10F. After 16 h of incubation in 37 °C, the occurrence of amplified 16S rRNA genes was confirmed through diagnostic PCR reactions with primers: pJET For and pJET Rev. (Thermo Scientific). For each species, 50 positive colonies were subjected to restriction fragment analysis (RFLP) using the *MspI* restrictive enzyme (Thermo Scientific). After this, selected colonies were incubated in liquid LB media with ampicillin (A&A Biotechnology) and then plasmids were isolated using Plasmid Mini AX Kit (A&A Biotechnology) and sequenced. Molecular cloning was repeated for each of the species examined. Additionally, 20 specimens of *M. pini* and 20 specimens of *O. urticae* were screened for the presence of *Wolbachia* (*M. pini*), as well as *Wolbachia*- and *Sodalis*-like (*O. urticae*) symbionts, using PCR reactions with *Wolbachia*- and *Sodalis*-specific primers (Fukatsu and Nikoh 1998; Zhou et al. 1998) under the following conditions: an initial denaturation step at 94 °C for a duration of 3 min, followed by 33 cycles at 94 °C for 30 s, 55 °C (*Wolbachia*) or 54 °C (*Sodalis*) for 40 s, 70 °C for 1 min 40 s, and a final extension step of 5 min at 72 °C. The PCR products were made visible through electrophoresis in 1.5% agarose gel stained with Midori Green (Nippon Genetics Europe), and subsequently sequenced (Genomed). The nucleotide sequences obtained were deposited into the GenBank database under the following accession numbers: MK462262–MK462265.

### Phylogenetic analyses

The phylogenetic analysis was performed based on sequences of 16S rRNA gene of *S. gorodetskia* symbiont and selected representatives of the Alphaproteobacteria phylum. The sequences, homologous to the sequence obtained, were found in the GenBank database using CLC MainWorkbench 7 software. The sequences were then edited using BioEdit Sequence Alignment Editor 5.0.9 (Hall 1999), and following this, the sequence alignments were generated using ClustalX 1.8 (Thompson et al. 1997). The base composition was estimated using MEGA 7.0. software (Kumar et al. 2016). The phylogenetic analysis was conducted by maximum likelihood (ML) and neighbor joining (NJ) methods using MEGA 7.0. software (Kumar et al. 2016).

### Light (LM) and electron microscopy (TEM)

The dissected abdomens of females of *O. urticae*, *S. gorodetskia*, and *M. pini* were fixed in 2.5% glutaraldehyde in 0.1 M phosphate buffer (pH 7.4) at room temperature for a period of 3 months. The material was then rinsed in the same buffer with an addition of sucrose (5.8 g/100 mL), postfixed for 1.5 h in 1% osmium tetroxide, dehydrated in a graded series of ethanol and acetone, and embedded in epoxy resin Epon 812 (Serva, Heidelberg, Germany). Semithin sections were stained with 1% methylene blue in 1% borax and photographed under a Nikon Eclipse 80i light microscope. Ultrathin sections were contrasted with uranyl acetate and lead citrate and examined in a JEM 100 SX EM and Jeol JEM 2100 transmission electron microscopes at 80 kV.

## Results

**Fig. 1:**
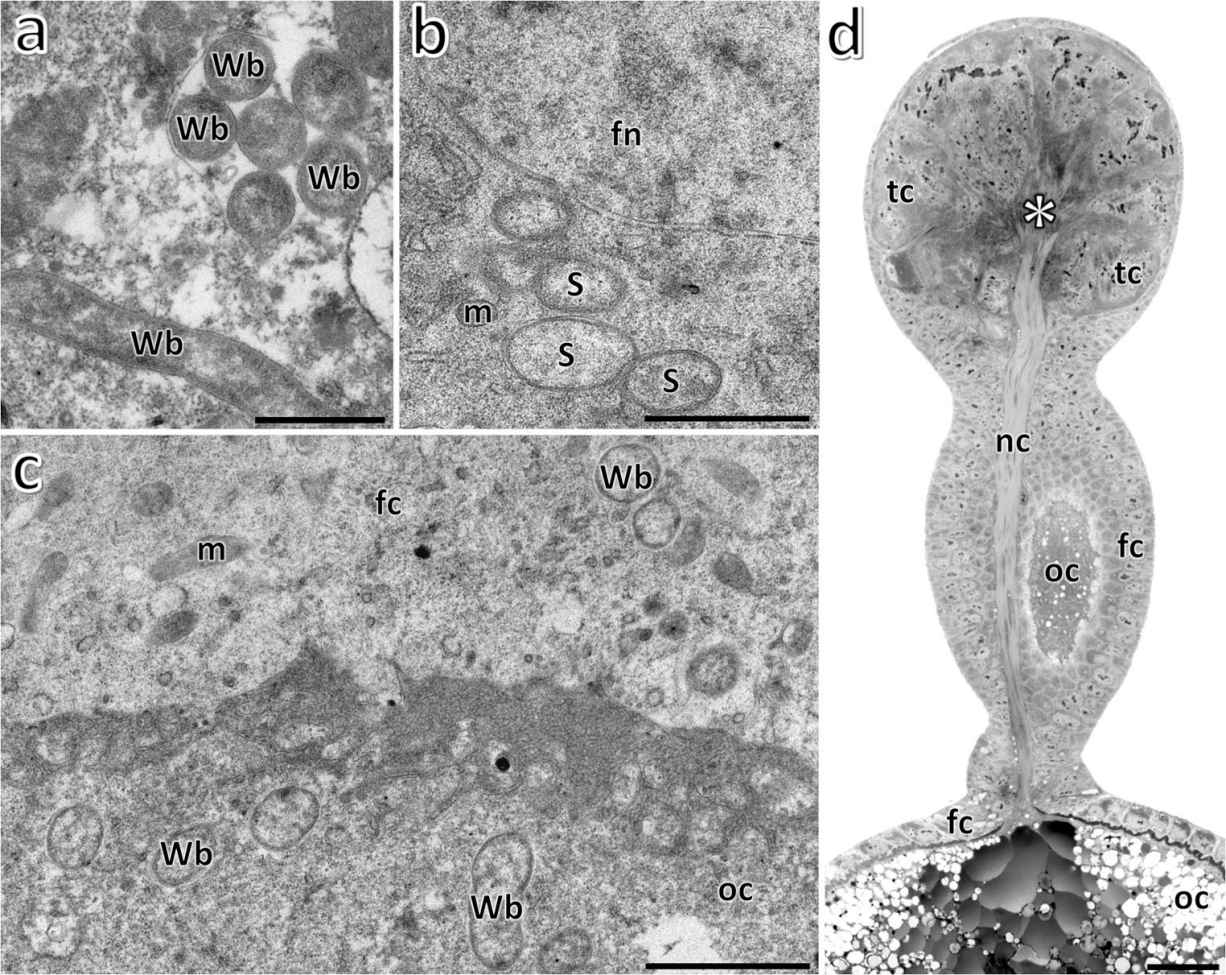
Distribution of symbiotic bacteria in *Orthezia urticae* (Ortheziidae). **a** Bacteria *Wolbachia* (Wb) in the cytoplasm of fat body cell. TEM, scale bar = 1 μm. **b** Bacteria *Sodalis* (S) in the cytoplasm of follicular cell. Follicular cell nucleus (fn); mitochondrium (m). TEM, scale bar = 2 μm. **c** Bacteria *Wolbachia* (Wb) in the cytoplasm of the follicular cell (fc) and the oocyte (oc). Mitochondrium (m). TEM, scale bar = 1 μm. **d** Ovariole (longitudinal section). Follicular cells (fc); nutritive cord (nc); oocyte (oc); trophocyte (tc); trophic core (asterisk). LM, scale bar = 40 μm

Ultrastructural analyses revealed that in the fat body cells (Fig. 1a), gut epithelium, in the ovarioles (i.e., structural and functional units constituting insect ovaries), and cells of the lateral oviduct of all the examined specimens of *Orthezia urticae*, numerous small, rod-shaped bacteria with a mean diameter of 0.5 μm and length of 1.9 μm are present. The highest concentration of these bacteria has been observed in fat body and in ovarioles. Within the ovarioles, the bacteria are dispersed throughout all the cells: in follicular cells (Fig. 1c), oocytes (Fig. 1c) and trophocytes (not shown) (for the ovariole organization in scale insects, see Fig. 1d). In ovarioles of some specimens aside from small bacteria, large, elongated bacteria were observed (Fig. 1b). The latter are approximately 1 μm in diameter and are significantly less numerous than the small, rod-shaped bacteria. The comparison of ultrastructural and molecular results indicates that the smaller microorganisms represent bacteria *Wolbachia*, whereas the larger ones belong to the genus *Sodalis*. Screen PCR reactions using symbiont specific primers have revealed that bacteria *Wolbachia* occur in all 20 specimens examined, whereas *Sodalis*-like symbionts were detected only in 9 out of 20 individuals. The comparison of the obtained 16S rRNA gene sequences of *Wolbachia*, as well as *Sodalis*-like symbionts (respectively), has indicated that they are identical. The sequence of 16S rRNA gene of *Wolbachia* displays a high similarity (99%) to the 16S rRNA gene of bacteria *Wolbachia* occurring in the body of beetles belonging to the genus *Diabrotica* [AY007550, AY007548, AY007447]. In turn, the sequence of 16S rRNA gene of *Sodalis*-like symbiont detected in some specimens of *O. urticae* shows a high similarity (98%) to *Sodalis* bacteria associated with the weevil *Curculio hachijoensis* [AB746396].

**Fig. 2:**
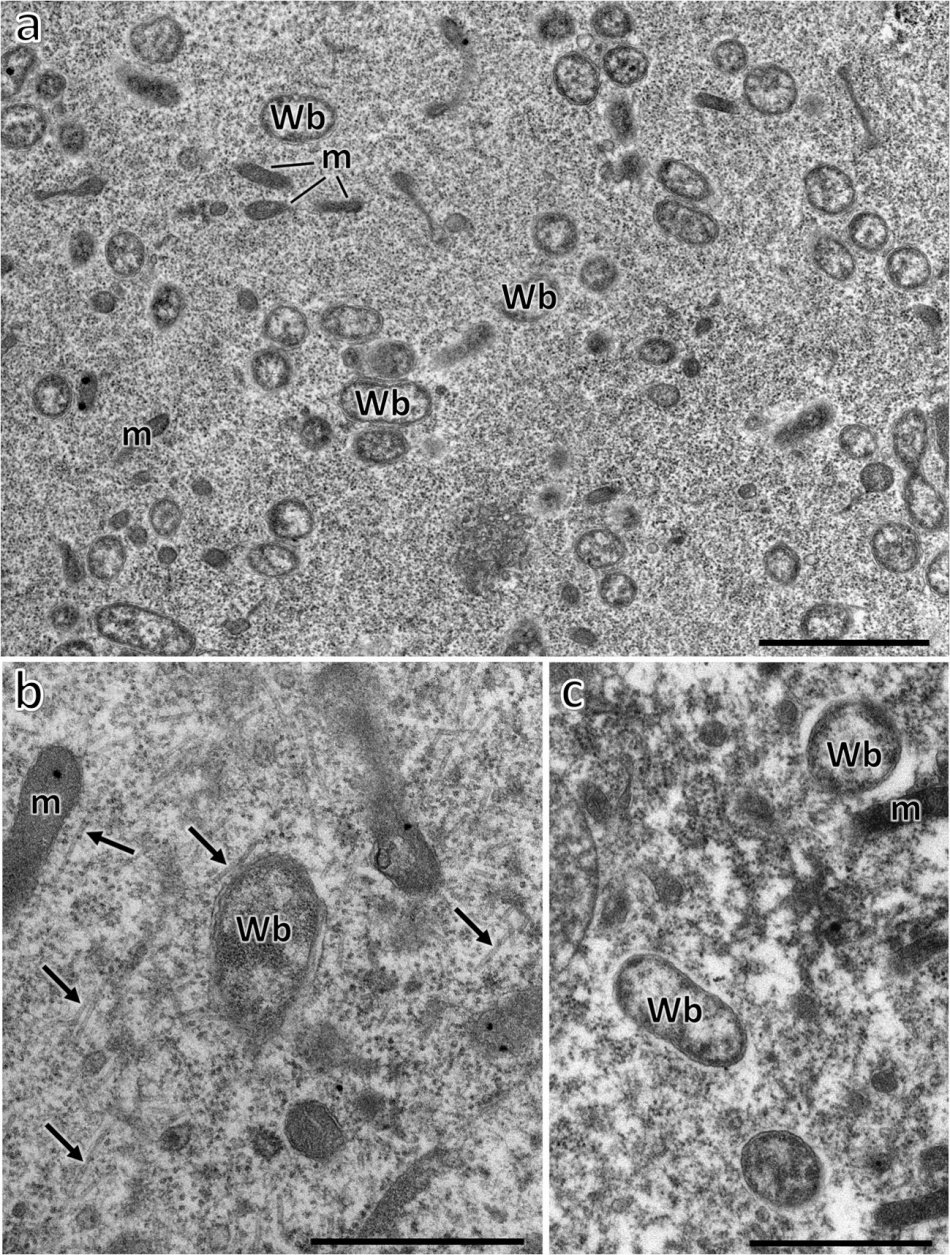
Distribution of symbiotic bacteria in *Matsucoccus pini* (Matsucoccidae). **a** Bacteria *Wolbachia* (Wb) in the trophocyte cytoplasm. Mitochondrium (m). TEM, scale bar = 2 μm. **b** Bacterium *Wolbachia* (Wb) migrating through the trophic core to the oocyte. Microtubules (arrows); mitochondrium (m). TEM, scale bar = 1 μm. **c** Bacteria *Wolbachia* (Wb) in the oocyte cytoplasm. Mitochondrium (m). TEM, scale bar = 1 μm

Ultrastructural observations indicated that in the body of *Matsucoccus pini*, small, rod-shaped bacteria measuring about 0.5 μm in diameter and 1 μm in length are present (Fig. 2a–c). Just as in *O. urticae*, the bacteria are distributed in different organs: in the fat body cells, in gut epithelium, and in the ovaries (Fig. 2a–c). Since molecular analyses showed that in the body of *M. pini*, only bacteria *Wolbachia* are present, the small, rod-shaped microorganisms represent this species. All the obtained sequences of 16S rRNA genes of examined individuals of *M. pini* are identical and show a 99% similarity to the homologous sequences isolated from *Drosophila incompta* [CP011148] and *Drosophila simulans* [CP001391].

**Fig. 3:**
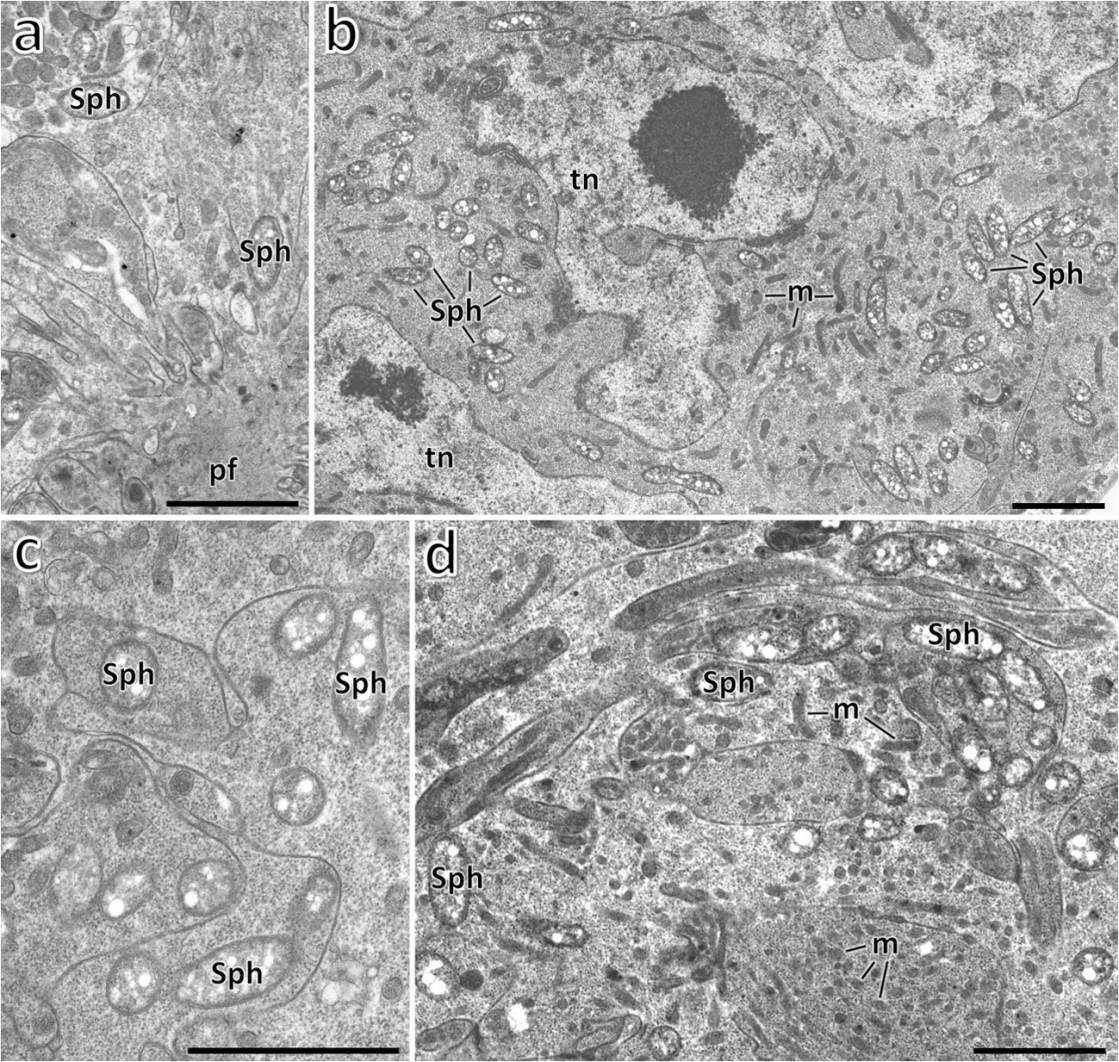
Distribution of symbiotic bacteria in *Steingelia gorodetskia* (Steingeliidae). **a** Bacteria *Sphingomonas* (Sph) in the cytoplasm of cystocytes in the ovary of the last instar larva. Polyfusome (pf). TEM, scale bar = 2 μm. **b** Bacteria *Sphingomonas* (Sph) in the cytoplasm of trophocyte of the adult female. Mitochondrium (m); Trophocyte nucleus (tn). TEM, scale bar = 2 μm. **c** Bacteria *Sphingomonas* (Sph) in processes of trophocytes during the migration via the trophic core and the nutritive cord to the oocyte. TEM, scale bar = 2 μm. **d** Bacteria *Sphingomonas* (Sph) migrate through the trophic core to the oocyte. Mitochondrium (m). TEM, scale bar = 2 μm

**Fig. 4:**
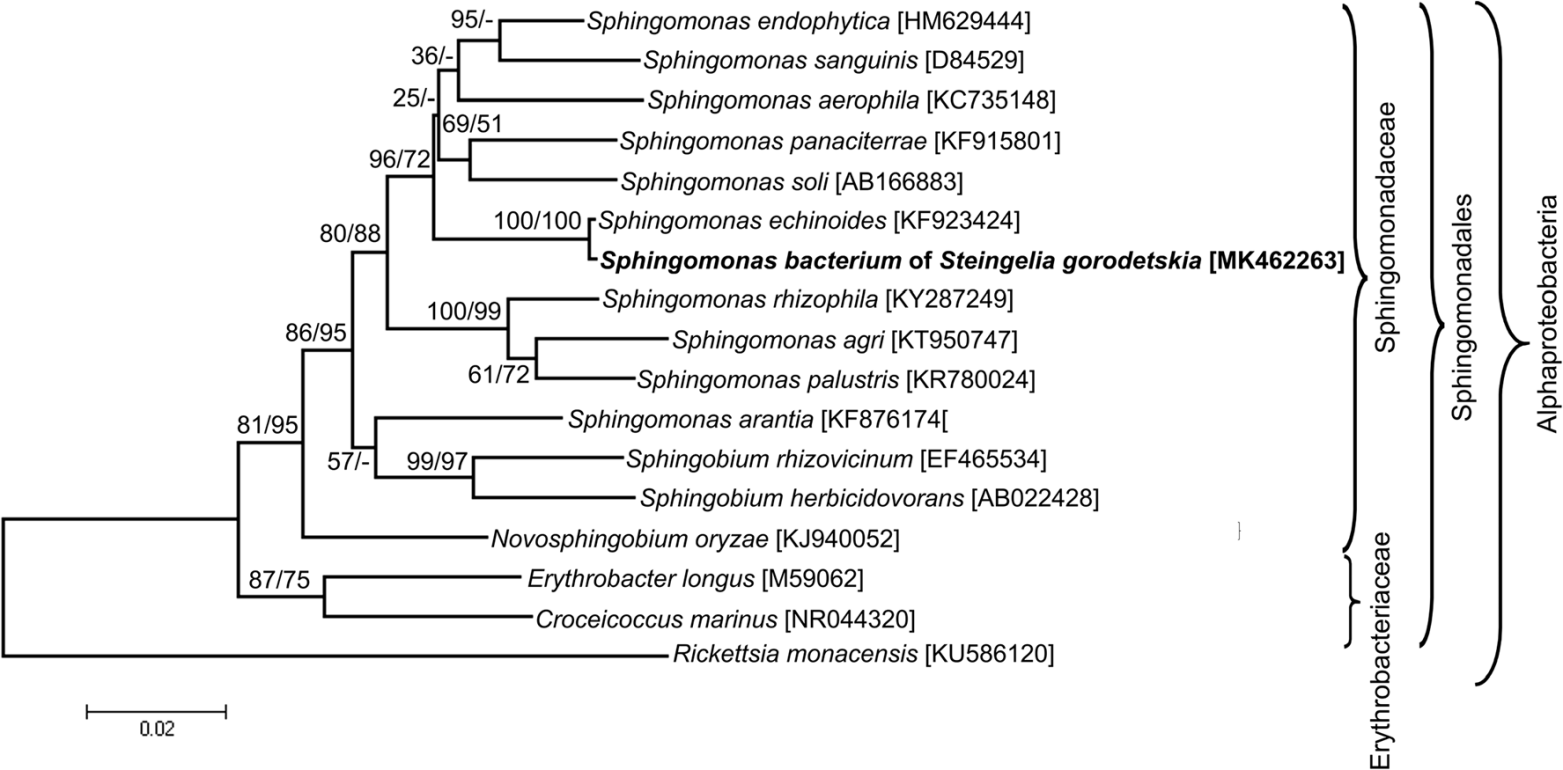
Phylogenetic tree based on 16S rRNA genes showing the systematic affiliation of bacterial symbiont of *Steingelia gorodetskia*. The numbers indicate the neighbor joining and maximum likelihood bootstrap values, respectively (in brackets, GenBank accession numbers of 16S rRNA genes)

Numerous small, rod-shaped bacteria are present in the fat body cells, the gut epithelium, ovaries, and the cells of the lateral oviduct of all the individuals of *Steingelia gorodetskia* (Fig. 3a–d) that were collected in two different locations. The highest amount of these bacteria was observed in the ovary: in the cystocytes (i.e., undifferentiated germ cells) constituting larval ovaries (Fig. 3a) as well as in the ovarioles of adult females (Fig. 3b–d): in trophocytes, in the cells of the inner epithelial sheath, in oocytes, in follicular cells surrounding the developing oocytes, in trophic core and nutritive cords. These microorganisms possess several electron-translucent areas within the cytoplasm and have measurements of about 0.4 μm in diameter and 1.4 μm in length. Neither the ultrastructural observations nor the molecular analyses revealed the presence in the body of *S. gorodetskia* of other species of bacteria. Molecular phylogenetic analyses based on the sequence of 16S rRNA gene indicated that bacteria present in *S. gorodetskia* belong to the Sphingomonadales order within Alphaproteobacteria phylum and are closely related to the soil bacterium *Sphingomonas echinoides* (Fig. 4). The length of the sequences used in the phylogenetic analysis was 1297 bp, whereas the composition of the nucleotide was approximately equal: 21.2% T, 23.1% C, 24.8% A, and 30.9% G.

The presence of bacteria (*Wolbachia* and *Sodalis* in *O. urticae*, *Wolbachia* in *M. pini*, *Sphingomonas* in *S. gorodetskia*) in the ovarioles of all the examined individuals indicates that these microorganisms are transovarially transmitted from one generation to the next. The bacteria to reach the oocyte migrate from trophocytes through the trophic core and nutritive cords (Figs. 2b, c and 3c, d) (for further details concerning ovary organization in Ortheziidae, Steingeliidae, and Matsucoccidae, see Szklarzewicz and Biliński 1995; Szklarzewicz 1997; Koteja et al. 2003; Szklarzewicz et al. 2014).

## Discussion

Our molecular and detailed ultrastructural analyses showed that members of archaeococcoid scale insects: *Orthezia urticae*, *Matsucoccus pini*, and *Steingelia gorodetskia*, are host to bacterial associates. It should be stressed that *M. pini* and *S. gorodetskia* were regarded by Buchner (1966) as asymbiotic. Koteja et al. (2003), who were the first to find small, rod-shaped bacteria residing in all the cells constituting the ovarioles of *S. gorodetskia*, proposed several explanations of this discrepancy between their’s and Buchner’s observations, e.g., the bacteria were too small to be detected under light microscope, the observed bacteria occur in some populations of *S. gorodetskia* only, these microorganisms do not represent symbionts. Our results have resolved some of these questions; however, some of them remain still open. Both ultrastructural and molecular analyses of individuals collected in different years and different localities clearly indicated that there is no doubt that the bacteria *Sphingomonas* are present in all members of the species *S. gorodetskia.* Thus, it seems probable that due to the small size of these bacteria, Buchner was unable to observe them using paraffin technique. The function of these bacteria remains unclear; however, the lack of any symptoms of their destructive influence on growth and reproduction of *S. gorodetskia* (Koteja and Żak-Ogaza 1981; Koteja et al. 2003, this study) suggests the positive impact of *Sphingomonas* on these insects. This in turn leads us to the hypothesis that the bacterium *Sphingomonas* may function as the symbiont of *S. gorodetskia.* However, in order to determine the exact role of this bacterium, further genomic studies are required. It should be stressed that some features of this cohabitation, such as a lack of specialized bacteriocytes and distribution of the bacteria *Sphingomonas* in different internal organs of *S. gorodetskia*, indicate its very young condition. The symbionts of most scale insects are harbored in specialized bacteriocytes (Buchner 1965); however, in some species, e.g., *Acanthococcus aceris* and *Gossyparia spuria* (both belonging to family Eriococcidae), symbiotic bacteria *Burkholderia* do not occupy bacteriocytes, but are dispersed in fat body cells (Michalik et al. 2016).

The bacteria belonging to the genus *Sphingomonas* are free living microorganisms widely distributed in the environment (in fresh and sea water, in soil); however, some of them, such as, e.g., *Sphingomonas paucimobilis*, may be the cause of diseases in humans and animals (White et al. 1996; Takeuchi et al. 2001; Ryan and Adley 2010; Feng et al. 2014). To our knowledge, the bacterium *Sphingomonas* has not been reported as the intracellular symbiont of insects so far. Tang et al. (2010), who examined three populations of the brown planthopper, *Nilaparvata lugens*, detected alphaproteobacteria related to *Sphingomonas* in only two of them. Thus, the occurrence of the bacterium *Sphingomonas* in only some of the examined individuals indicated that it cannot be an obligate symbiont of *Nilaparvata lugens.*

Our molecular phylogenetic analyses have revealed that the bacterium *Sphingomonas* present in *S. gorodetskia* is closely related to *Sphingomonas echinoides* which, like other species of the genus *Sphingomonas*, commonly occurs in the soil (Takeuchi et al. 2001). This finding suggests that the bacteria residing in cells of all the individuals of *S. gorodetskia* are descendants of free living soil bacterium which has been acquired by an ancestor of these insects. It seems that the transition of bacterium *Sphingomonas* from free living to symbiotic status occurred according to the same evolutionary scenario as the acquisition of bacterium *Burkholderia* through eriococids *A. aceris* and *G. spuria* (Michalik et al. 2016). The bacteria of the genus *Burkholderia*, similarly to bacteria *Sphingomonas*, commonly occur in the soil and may be also plant, animal, and human pathogens (Stoyanova et al. 2007). It is worth mentioning that apart from the two species of eriococcids mentioned above, the symbiotic bacterium *Burkholderia* has also been detected in the crypts of the midgut of some stinkbugs (Kikuchi et al. 2005, 2007, 2011; Kikuchi and Fukatsu 2008; Itoh et al. 2014). Moreover, Kikuchi et al. (2005) and Itoh et al. (2014) have shown that the broad-headed stinkbug, *Rhiptortus clavatus*, and oriental chinch bug, *Cavelerius saccarivorus*, may transmit the bacterium *Burkholderia* between generations both vertically and horizontally (i.e., through the acquisition of these microorganisms by each generation directly from the environment). Kikuchi et al. (2007, 2011) have also revealed that symbiotic bacteria *Burkholderia* isolated from the midgut crypts of stinkbugs, in contrast to the bacteriocyte-associated symbionts of other hemipterans, grow on standard bacterial media. Thus, the observations mentioned above strongly support the view that symbioses of hemipterans and bacteria *Burkholderia* are at a much earlier stage than bacteriocyte symbioses in other insects. It should also be mentioned that the assumption that the bacterium *Sphingomonas* in *S. gorodetskia* is a descendant of the soil bacterium corresponds well with Koteja’s (1985) hypothesis, which maintains that an enormous diversity of symbionts associated with scale insects results from the permanent contact of ancestors of those insects with the soil bacteria in forest litter (i.e., the primary habitat of these insects). According to Koteja (1985), the groups of scale insects which have already diverged changed their feeding behavior from saprophagic into plant sap-sucking at a different time. Since the “new diet” required the support of symbionts, the particular groups of scale insects acquired different symbionts. It should be stressed that recent molecular analyses which show that the symbionts of scale insects belong to different bacterial taxa (von Dohlen et al. 2001; Thao et al. 2002; Gruwell et al. 2007, 2010, 2014; Kono et al. 2008; Matsuura et al. 2009; Ramirez-Puebla et al. 2010; Gatehouse et al. 2011; Dhami et al. 2012; Rosenblueth et al. 2012; Koga et al. 2013a; Rosas-Pérez et al. 2014; Michalik et al. 2016, 2018; Szabo et al. 2017; Szklarzewicz et al. 2018) strongly support this hypothesis.

Another argument supporting the assumption on the young condition of symbiosis in *S. gorodetskia* concerns the mode of the transmission of bacteria from mother to progeny. Both the results of earlier observations of Koteja et al. (2003) and present studies indicate that, in contrast to scale insects which are characterized by a long-lasting symbiosis with bacterial associates (i.e., representing “bacteriocyte symbiosis”), the females of *S. gorodetskia* have not yet developed a specialized mode of symbiont transmission. It is commonly known that different groups of scale insects are characterized by diverse methods of symbiont transmission (Buchner 1965; Szklarzewicz and Michalik 2017). In most scale insects, symbionts infect ovaries which contain oocytes during the advanced stage of vitellogenesis, e.g., in members of the family Pseudococcidae and Eriococcidae examined thus far, in *Puto superbus* (Putoidae), bacteria invade the anterior pole of the vitellogenic ovariole, whereas in *Palaeococcus fuscipennis* (Monophlebidae) and *Porphyrophora polonica* (Margarodidae) the posterior pole of the vitellogenic ovariole is infected (Buchner 1965, 1966; von Dohlen et al. 2001; Szklarzewicz et al. 2006, 2018; Michalik et al. 2016, 2018). In *Marchalina hellenica* (Marchalinidae) and *Puto albicans* (Putoidae), bacteria infect the undifferentiated germ cells (= cystocytes) constituting the larval ovaries (Szklarzewicz et al. 2010, 2013). Ultrastructural observations revealed that in *S. gorodetskia* none of the above mentioned modes of symbiont transmission occurs. It was observed that in the reproductive females of *S. gorodetskia*, bacteria *Sphingomonas* are dispersed in all the cells constituting the ovariole (in somatic cells, i.e., follicular cells and the cells of the ovariole sheath, as well as in germ cells, i.e., in oocytes and trophocytes). It seems probable that bacteria *Sphingomonas*, similarly to symbionts of *M. hellenica* and *P. albicans* (see above), infect the ovaries of the larvae before the differentiation of cystocytes into oocytes and trophocytes, however, apart from germ cells they also attack somatic cells. Since the symbiotic bacteria have to reach the oocyte (and, consequently, the next generation), they migrate from trophocytes to these cells via the trophic core and nutritive cord. Thus, ultrastructural observations indicate that in *S. gorodetskia* bacteria *Sphingomonas* are transovarially inherited; however, in not so specialized a way as in remaining scale insects. It is worth mentioning that bacteria *Burkholderia*, which represent the obligate symbionts of eriococcids *A. aceris* and *G. spuria*, in spite of their “less advanced” localization in fat body cells, are transmitted to the next generation in a mode typical for most hemipterans, i.e., through the infection of older oocytes (Michalik et al. 2016).

Both ultrastructural and molecular analyses have revealed that all the examined individuals of *M. pini* and *O. urticae* were colonized by numerous bacteria *Wolbachia*. The bacterium *Wolbachia* is widely distributed within insects, other arthropods, and nematodes (Werren 1997; Stouthamer et al. 1999; Werren et al. 2008). In most arthropods, bacterium *Wolbachia* is regarded as a “reproductive manipulator” or “reproductive parasite.” Since the bacterium *Wolbachia* is maternally inherited through the infection of oocytes, it developed several strategies of eliminating males, e.g., through the killing of male embryos, feminization of male embryos, cytoplasmic incompatibility in infected males and uninfected females, and the induction of parthenogenesis. So far, the nutritional, mutualistic relationship between *Wolbachia* and its host has been reported for filarial nematodes and bedbug *Cimex lectularius* only (Hosokawa et al. 2010; Slatko et al. 2010). Genomic analyses, as well as experiments with antibiotic treatment have shown that both in filarial nematodes and in *C. lectularius* bacterium *Wolbachia* plays an essential role for the proper growth and reproduction of the host: in filarial nematodes, the bacterium *Wolbachia* is responsible for heme biosynthesis (Slatko et al. 2010); in *C. lectularius*, this microorganism provides B vitamins (Hosokawa et al. 2010). Moreover, in *C. lectularius*, bacteria *Wolbachia* are harbored in specialized bacteriocytes, whereas in remaining insects these microorganisms are dispersed in different tissue. According to Nikoh et al. (2014), the situation observed in *C. lectularius* is an example of the evolutionary transition from facultative symbiosis to obligate nutritional mutualism.

There are only several reports on the occurrence of *Wolbachia* in scale insects (Duron et al. 2008; Matsuura et al. 2009; Vashishtha et al. 2011; Dhami et al. 2012; Szklarzewicz et al. 2018); however, the role of this bacterium in biology of examined scale insects remains still unknown. Our observations during the collection of *M. pini* and *O. urticae* in the field indicated that males are as numerous as the females. This, in turn, suggests that the bacterium *Wolbachia* does not negatively affect the number of males in the examined population of *M. pini* and *O. urticae*. Thus, to answer the question whether *Wolbachia* is beneficial for *M. pini* and *O. urticae* or if this bacterium represents only a guest microorganism, further studies are required.

It should be stressed that neither the use of ultrastructural nor molecular methods showed the presence of bacteria other than bacterium *Wolbachia* in the body of *M. pini*. The absence of other obligate symbionts in *M. pini* is probably related to the fact that these insects are parenchyma feeders (Siewniak 1976). Therefore, receiving nutritious food, scale insects of the *Matsucoccus* genus did not enter into symbiotic relationships with microorganisms supplementing their diet.

The presence of *Sodalis*-like bacteria in only some individuals of *O. urticae* suggests that this microorganism does not play a nutritional role in the biology of these insects. Taking into consideration the fact that *Sodalis*-like bacteria in numerous plant sap-sucking hemipterans represent “novel,” very expensive, obligate symbiont (Koga et al. 2013b; Koga and Moran 2014; Michalik et al. 2014; Vera-Ponce de León et al. 2016; Kobiałka et al. 2018a, b; Szklarzewicz et al. 2018), it is possible that the situation observed in *O. urticae* may be the beginning of the colonization of the scale insects of this species through *Sodalis*.

## Electronic supplementary material

Fig. S1 **a** Young adult female of *Orthezia urticae* (Ortheziidae) (photographed by Katarzyna Michalik). **b** Female of *Steingelia gorodetskia* (Steingeliidae) (photographed by Katarzyna Michalik). **c** Female of *Matsucoccus pini* (Matsucoccidae) (photographed by Marzena Zmarzły). Stereomicroscope, scale bar = 1 mm (PNG 5363 kb)

High resolution image (TIF 16220 kb)
