# Supplementary figures and images for: Bacterial associates of Orthezia urticae, Matsucoccus pini, and Steingelia gorodetskia - scale insects of archaeoccoid families Ortheziidae, Matsucoccidae, and Steingeliidae (Hemiptera, Coccomorpha)
Source: Protoplasma. 2019 Apr 17;256(5):1205–15. doi: 10.1007/s00709-019-01377-z (PMC6713686; doi:10.1007/s00709-019-01377-z)

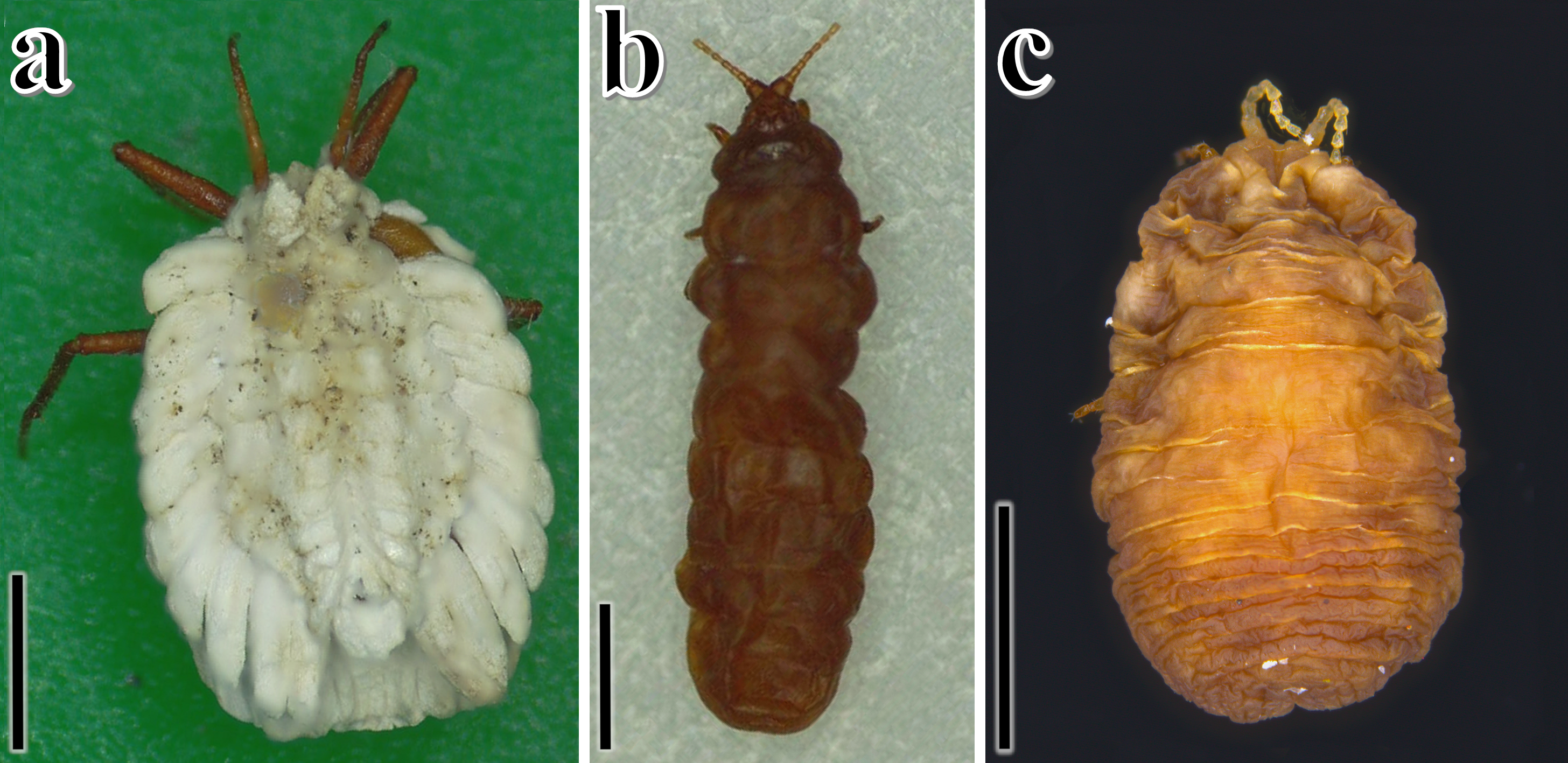

Supplement: Supplementary file 1 — a Young adult female of Orthezia urticae (Ortheziidae) (photographed by Katarzyna Michalik). b Female of Steingelia gorodetskia (Steingeliidae) (photographed by Katarzyna Michalik). c Female of Matsucoccus pini (Matsucoccidae) (photographed by Marzena Zmarzły). Stereomicroscope, scale bar = 1 mm (PNG 5363 kb) [file 709_2019_1377_Fig5_ESM.png]

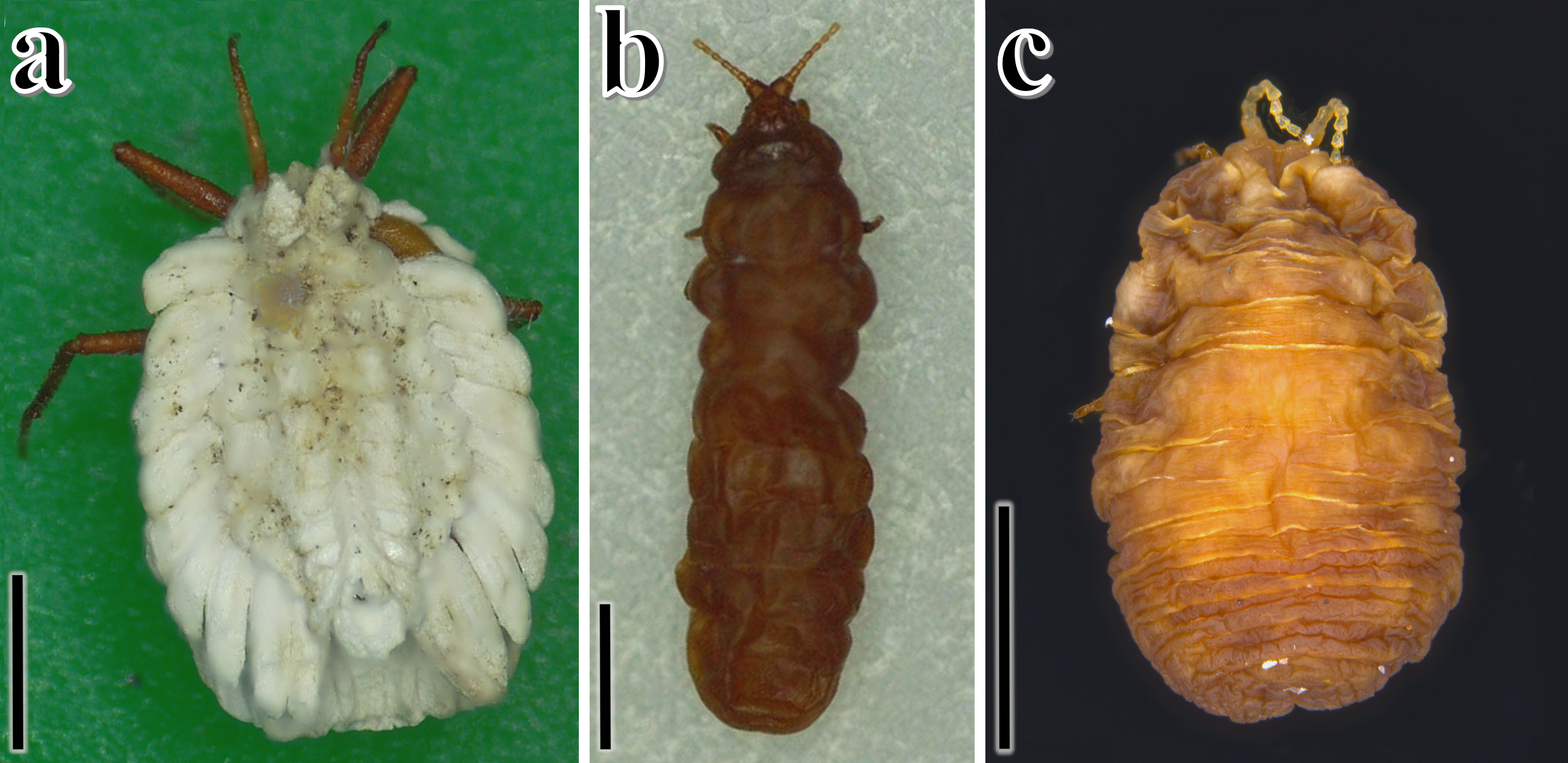

Supplement: Supplementary file 2 — High resolution image (TIF 16220 kb) [file 709_2019_1377_MOESM1_ESM.tif]
